# Appetite Suppression and Interleukin 17 Receptor Signaling Activation of Colonic Mycobiota Dysbiosis Induced by High Temperature and High Humidity Conditions

**DOI:** 10.3389/fcimb.2021.657807

**Published:** 2021-09-10

**Authors:** Yinrui Guo, Hongya Guo, Lingyan Qiu, Yuanfei Fu, Xiangxiang Zhu, Haiting Zhang, Jian Wang, Diling Chen

**Affiliations:** ^1^School of Basic Medical Science, Guangzhou University of Chinese Medicine, Guangzhou, China; ^2^The Fourth Clinical Medicine School, Guangzhou University of Chinese Medicine, Guangzhou, China; ^3^State Key Laboratory of Applied Microbiology Southern China, Guangdong Provincial Key Laboratory of Microbial Culture Collection and Application, Institute of Microbiology, Guangdong Academy of Sciences, Guangzhou, China; ^4^Academy of Life Sciences, Jinan University, Guangzhou, China; ^5^Department of Chinese Medicine, Guangdong Second Provincial General Hospital, Guangzhou, China

**Keywords:** colonic mycobiota, host appetite, gut-brain axis, climate change, immunity, IL-17R signaling

## Abstract

It is known that the microbiome affects human physiology, emotion, disease, growth, and development. Most humans exhibit reduced appetites under high temperature and high humidity (HTHH) conditions, and HTHH environments favor fungal growth. Therefore, we hypothesized that the colonic mycobiota may affect the host’s appetite under HTHH conditions. Changes in humidity are also associated with autoimmune diseases. In the current study mice were fed in an HTHH environment (32°C ± 2°C, relative humidity 95%) maintained *via* an artificial climate box for 8 hours per day for 21 days. Food intake, the colonic fungal microbiome, the feces metabolome, and appetite regulators were monitored. Components of the interleukin 17 pathway were also examined. In the experimental groups food intake and body weight were reduced, and the colonic mycobiota and fecal metabolome were substantially altered compared to control groups maintained at 25°C ± 2°C and relative humidity 65%. The appetite-related proteins LEPT and POMC were upregulated in the hypothalamus (*p* < 0.05), and *NYP* gene expression was downregulated (*p* < 0.05). The expression levels of PYY and O-linked β-N-acetylglucosamine were altered in colonic tissues (*p* < 0.05), and interleukin 17 expression was upregulated in the colon. There was a strong correlation between colonic fungus and sugar metabolism. *In fimo* some metabolites of cholesterol, tromethamine, and cadaverine were significantly increased. There was significant elevation of the characteristic fungi *Solicoccozyma aeria*, and associated appetite suppression and interleukin 17 receptor signaling activation in some susceptible hosts, and disturbance of gut bacteria and fungi. The results indicate that the gut mycobiota plays an important role in the hypothalamus endocrine system with respect to appetite regulation *via* the gut-brain axis, and also plays an indispensable role in the stability of the gut microbiome and immunity. The mechanisms involved in these associations require extensive further studies.

## Introduction

Our understanding of when and how abruptly this climate-driven disruption of biodiversity will occur is limited because biodiversity forecasts typically focus on individual snapshots of the future. It is certain that biodiversity is threatened by climate change. Climate change can have adverse effects on biodiversity, by shifting species distributions (Barnosky et al., 2012; Wernberg et al., 2016; Provost et al., 2017), increasing extinction rates (Hultman et al., 2015), altering breeding times (Miller et al., 2018; Lv et al., 2020), and changing plant growth periods (Piao et al., 2019).

An increasing number of reports indicate that climate change effects the microbial diversity in soil, forests, and oceans (Sen and Samanta, 2015; Ladau et al., 2018; Malard and Pearce, 2018; Praeg et al., 2019). Recent research suggests that gut microbes are involved in nearly every aspect of our lives, from nutrition (Gentile and Weir, 2018), to behavior (O’Toole and Jeffery, 2015; Torres-Fuentes et al., 2017; Strandwitz, 2018), to diseases and mental health (Jiang et al., 2017; Quigley, 2017; Brial et al., 2018; Strandwitz, 2018). Studies on gut microflora have generally focused on the characteristics of bacteria, and ignored the potential effects of fungi on metabolic health.

Although fungi are only a small subset of microbes, they are very important in homeostatic balance (Li et al., 2019; Richard and Sokol, 2019). Many fungi may serve as a pathogen reservoir, and also play a key role in maintaining the functioning of the gut microbiota (Leonardi et al., 2018; Jones et al., 2019; Zhang et al., 2020). Specifically, *Malassezia* spp. was enriched markedly in both mice and humans. Ablation of the mycobiome was protective against tumor growth in slowly progressive and invasive models of pancreatic ductal adenocarcinoma, and repopulation with a *Malassezia* species accelerated oncogenesis (Aykut et al., 2019; Wolf et al., 2020). Thus, it is very important to determine the composition and function of intestinal fungi that influence human health.

Temperature and humidity have been linked to almost all human diseases, such as influenza (Sooryanarain and Elankumaran, 2015), cardiovascular mortality (Zeng et al., 2017), chronic obstructive pulmonary disease (Mu et al., 2017), allergic rhinitis (Duan et al., 2019), and arthritis (Bai et al., 2012; Bossema et al., 2013; Beukenhorst et al., 2020), among others (Onozuka and Hashizume, 2011; Balato et al., 2013; Yang et al., 2017). Infectious diseases such as coronavirus disease 2019 and H7N9 are evidently particularly affected by temperature and humidity (Zhang et al., 2015; Yao et al., 2020). A study using a guinea pig model provided direct experimental evidence supporting the role of weather conditions in the dynamics of influenza, thereby addressing a long-standing question fundamental to the understanding of influenza epidemiology and evolution (Lowen et al., 2007; Sooryanarain and Elankumaran, 2015). Potential links between *Candida auris* and climate change (Hofer, 2019) and many skin diseases (Balato et al., 2013; Balato et al., 2014) have been suggested. In traditional Chinese medicine the climatic factors of season, weather, wind, temperature, and humidity are comprehensively considered in the diagnosis of human diseases. Climatic temperature and humidity can substantially affect pathogenic microorganisms and the occurrence and development of diseases related to them. In the present study temperature and humidity were investigated with the aim of generating experimental data on the effects of climatic factors on microorganisms, particularly fungi, and host health.

## Methods

### Animals and Treatments

Male C57BL/6 mice weighing 14–16 g and aged 28–35 days were purchased from the Center for Laboratory Animals, Guangdong Province (Certification number SCXK[Yue]2018-0002, **Table 1**). They were maintained in a temperature-controlled (25°C) and humidity-controlled (55% ± 10%) room at a 12-h light–dark cycle in an ordinary clean environment within the Guangdong Institute of Microbiology. Food and water were available to the mice. The Animal Ethics Committee of the Guangdong Institute of Microbiology approved all experimental protocols. All efforts were made to minimize the number of mice used, and their suffering.

**Table 1:** Experimental Model: Organisms/Strains.

Reagent or Resource	Source	Identifier	Official website
Mouse:C57BL/6	the Center of Laboratory Animals of Guangdong Province (SCXK [Yue] 2008-0020, SYXK [Yue] 2008-0085	N/A	http://www.gdmlac.com.cn/

#### Model 1

**Figure 1:**
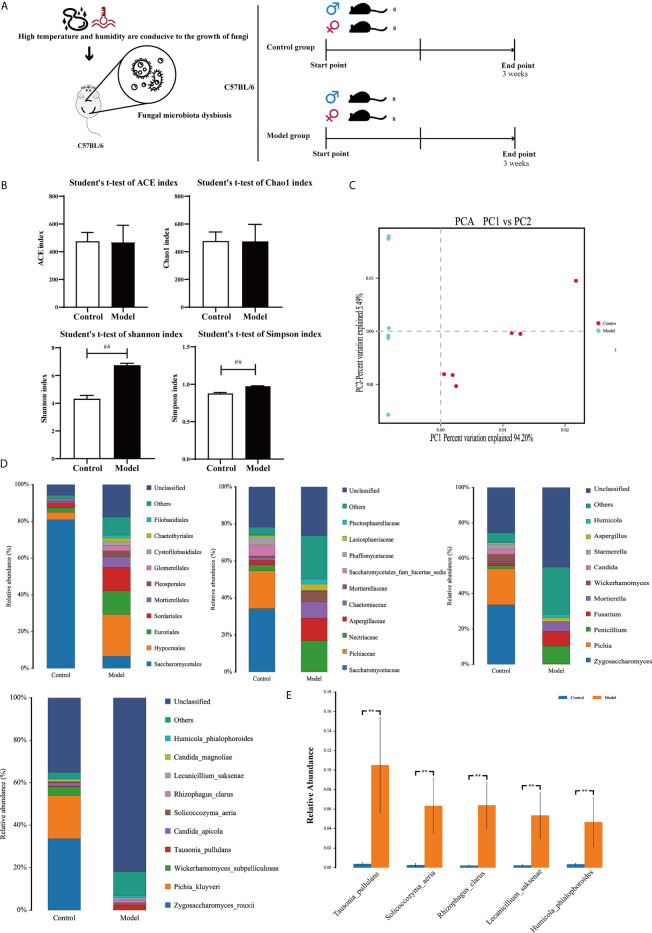
Colonic mycobiota dysbiosis under HTHH conditions **(A)** Schematic diagram of the HTHH model. **(B)** Alpha diversity analysis of colonic fungal microbiota, and the beta diversity analysis effects ACE, the Chao1 estimator, Simpson’s diversity index, and the Shannon diversity index. **(C)** Principal component analysis. **(D)** Histogram of microbe distributions at the order, family, genus, and species levels. Only the top ten most abundant species are individually shown, and the additional microbes are combined as “Others”. “Unclassified” represents species that have not been taxonomically annotated. **(E)** Analysis of variance between groups at the species level, selecting five with the greatest changes in abundance.

Mice were randomly divided into two groups of 16; an experimental group and a control group. The control group was not treated. The experimental group was exposed to high temperature and high humidity (HTHH) conditions (temperature 32°C ± 2°C, relative humidity 95%) *via* an artificial climate box (**Figure 1A**).

#### Model 2

Mice were randomly divided into two groups of 16, the treatment group, and the control group. The treatment groups were generated by gavage (ig) of 0.4 mL *Solicoccozyma aeria* (7.0 × 10^8^ colony-forming units [CFU]/mL) once a day for 2 weeks. The control group was generated by gavage (ig) of 0.4 mL distilled water once a day for 2 weeks (**Figure 4A**).

### Saliva Collection From Humans During Warm and Humid Conditions

From May to June in Guangdong in 2020, volunteers with a lack of appetite and a greasy tongue coating due to warm and humid conditions were recruited. According to our long-term observations, people living in humid conditions or warm and humid conditions tend to exhibit a thick and greasy tongue coating.

### Microbiome ITS and 16S rDNA Analysis

Colonic digesta were collected after mice were killed, then frozen in liquid nitrogen. Saliva was collected in a tube with a DNA-protective fluid. Total DNA was extracted from 250–500mg of sample.

### Construction and Sequencing

Primers were designed based on the conservative regions of the genes of interest, and a sequencing connector was added to the ends of the primers. Microbial 16S rDNA genes were amplified using the forward primer 338F 5’-ACTCCTACGGGAGGCAGCA-3’ and the reverse primer 806R 5’-GGACTACHVGGGTWTCTAAT-3’. Microbial ITS genes were amplified using the forward primer ITS1F 5’-CTTGGTCATTTAGAGGAAGTAA-3’ and the reverse primer ITS2 5’-GCTGCGTTCTTCATCGATGC-3’. PCR amplification was performed and the products were purified, quantified, and homogenized to form a sequencing library. The library was sequenced *via* an Illumina HiSeq 2500. The original image data files obtained *via* high-throughput sequencing (Illumina HiSeq and other sequencing platforms) were analyzed and converted into original sequencing reads by Base Calling, and the results were stored in FASTQ (fq) file format, which contained the sequence information and the corresponding sequencing quality information. Data is stored in SRA and the access can be found in **Table 2**.

**Table 2:** Deposited Data.

Resource	Description	Identifier
PRJNA693676	Changes of colonic fungal microbiome in C57BL/6 mice under warm and humid environment (T=32 ± 2 °C, RH=95%)	SRA
PRJNA694096	Fungal microbiome in colon of C57BL/6J mice by gavage Solicoccozyma aeria	SRA
PRJNA694055	Bacterial microbiome in colon of C57BL/6J mice by gavage Solicoccozyma aeria	SRA
PRJNA690659	Changes of human saliva fungal microbiomes in high temperature and high humidity environment	SRA

### GC-MS Fecal Metabolomics Analysis

Each 40-mg feces sample was homogenized in 400 μL deionized water containing 10 μg/mL L-norvaline as an internal standard. After centrifugation at 14,000 *g* and 4°C for 15 min, 300 μL of supernatant was transferred. The extraction was repeated by adding 600 μL of ice-cold methanol to the residue. The supernatants from two extractions were combined. A 400-μL sample of combined supernatants and 10 μL of internal standard solution (50 μg/mL of L-norleucine) were combined and evaporated to dryness under a nitrogen stream. The residue was reconstituted in 30 μL of 20 mg/mL methoxyamine hydrochloride in pyridine, and the resulting mixture was incubated at 37°C for 90 min. A 30-μL aliquot of BSTFA with 1% TMCS was added to the mixture and derivatized at 70°C for 60 min prior to GC-MS metabolomics analysis.

Metabolomics instrumental analysis was performed using an Agilent 7890A gas chromatography system coupled with an Agilent 5975C inert MSD system (Agilent Technologies Inc., CA, USA). An Optima^®^ 5 MS Accent fused-silica capillary column (30 m × 0.25 mm × 0.25 μm; Macherey-Nagel, Düren, Germany) was utilized to separate the derivatives. Helium (> 99.999% pure) was used as a carrier gas at a constant flow rate of 1 mL/min through the column. The injection volume was 1 μL in split mode (2:1), and the solvent delay time was 6 min. The initial oven temperature was 70°C for 2 min, then it was increased to 160°C at a rate of 6°C per min, then to 240°C at a rate of 10°C per min, then to 300°C at a rate of 20°C per min, and lastly it was held at 300°C for 6 min. The temperatures of the injector, transfer line, and electron impact ion source were set to 250°C, 260°C, and 230°C, respectively. The electron ionization energy was 70 eV, and data were collected in full scan mode (m/z 50–600).

### Correlational Analysis of Microbiome Metabolomics

Spearman’s correlation conjoint analysis of different colonic fungal genera and different fecal metabolites was performed. R was used to generate a heat map. Red represents positive correlations and blue represents negative correlations. Based on the results of the correlational analysis, correlation networks were constructed by selecting *p* values < 0.01 and correlation coefficients > 0.7 using Cytoscape software.

### Histopathology and Immunostaining

Colonic tissues were removed and fixed in 4% paraformaldehyde at pH 7.4 for pathological observation. The samples were then washed, dehydrated, transparency, dipped in paraffin wax, and embedded, then 3-μm sections were generated. Immunostaining and a two-step peroxidase conjugated polymer technique (DAKO Envision kit, DAKO, Carpinteria, CA, USA) were applied, then the slides were observed *via* light microscopy.

### Western Blotting

Briefly, colon and hypothalamus tissue were dissected from mice and proteins were extracted with radioimmunoprecipitation assay lysis buffer. The proteins were separated *via* sodium dodecyl sulfate-polyacrylamide gel electrophoresis and transferred onto polyvinylidene fluoride membranes. After blocking with 5% skim milk in Tris-buffered saline (20 mM Tris-HCl, 500 mM NaCl, pH 7.4) with 0.2% Tween-20 (Aladdin, T104863) the membranes were probed with antibodies overnight at 4°C, followed by incubation with a horseradish peroxidase-conjugated goat anti-mouse (Servicebio, G2211-1-A) or goat anti-rabbit (Servicebio, G2210-2-A) secondary IgG antibody (1:2000). The primary antibodies and the reagents used were found in **Tables 3** and **4**.

**Table 3:** Antibodies.

Reagent or Resource	Source	Identifier	Working concentration	Official website
Leptin Receptor Antibody - C-terminal	Affinity	DF7139	1:2000	http://www.affbiotech.com/
POMC Antibody - C-terminal	Affinity	DF7154	1:1500	http://www.affbiotech.com/
IL17A Antibody - Internal	Affinity	DF6127	1:1000	http://www.affbiotech.com/
Peptide YY Polyclonal Antibody	proteintech	24294-1-AP	1:500	http://www.ptgcn.com/
Anti-IL-23 antibody	Abcam	ab45420	1:1000	https://www.abcam.com/
Anti-O-GlcNAc mouse mAb	PTM BIO	PTM-952	1:1000	http://www.ptm-biolab.com.cn/

**Table 4:** Chemicals, Reagent kit, Peptides, and Recombinant Proteins.

Reagent or Resource	Source	Identifier	Official website
RNAiso Plus	Takara	9109	http://www.takarabiomed.com.cn/
DNAiso Reagent	Takara	9770Q	http://www.takarabiomed.com.cn/
PrimeScript™ RT reagent Kit with gDNA Eraser (Perfect Real Time)	Takara	RR047A	http://www.takarabiomed.com.cn/
TB Green^®^ Premix Ex Taq™ II (Tli RNaseH Plus)	Takara	RR820A	http://www.takarabiomed.com.cn/
Glycogen Periodic Acid Schiff (PAS/Hematoxylin) Stain Kit	Solarbio	G1281	http://www.solarbio.com/index.php
Mouse IL-17(Interleukin 17) ELISA Kit E-EL-M0047c	Elabscience	E-EL-M0047c	https://www.elabscience.cn/
Saliva DNA Storage Tube	cwbio	CW2667M	https://www.cwbiosciences.com/home
T-PER™ Tissue Protein Extraction Reagent	Thermo Scientific™	78510	https://www.thermofisher.com/cn/zh/home.html

### Transcriptome Analysis

RNA concentration and purity was measured using NanoDrop 2000 (Thermo Fisher Scientific, Wilmington, DE). RNA integrity was assessed using the RNA Nano 6000 Assay Kit of the Agilent Bioanalyzer 2100 system (Agilent Technologies, CA, USA). A total amount of 1 μg RNA per sample was used as input material for the RNA sample preparations. Sequencing libraries were generated using NEBNext UltraTM RNA Library Prep Kit for Illumina (NEB, USA) following manufacturer’s recommendations and index codes were added to attribute sequences to each sample. Briefly, mRNA was purified from total RNA using poly-T oligo-attached magnetic beads. Fragmentation was carried out using divalent cations under elevated temperature in NEBNext First Strand Synthesis Reaction Buffer (5X). First strand cDNA was synthesized using random hexamer primer and M-MuLV Reverse Transcriptase. Second strand cDNA synthesis was subsequently performed using DNA Polymerase I and RNase H. Remaining overhangs were converted into blunt ends *via* exonuclease/polymerase activities. After adenylation of 3’ ends of DNA fragments, NEBNext Adaptor with hairpin loop structure were ligated to prepare for hybridization. In order to select cDNA fragments of preferentially 240 bp in length, the library fragments were purified with AMPure XP system (Beckman Coulter, Beverly, USA). Then 3 μl USER Enzyme (NEB, USA) was used with size-selected, adaptor-ligated cDNA at 37°C for 15 min followed by 5 min at 95°C before PCR. Then PCR was performed with Phusion High-Fidelity DNA polymerase, Universal PCR primers and Index (X) Primer. At last, PCR products were purified (AMPure XP system) and library quality was assessed on the Agilent Bioanalyzer 2100 system. The clustering of the index-coded samples was performed on a cBot Cluster Generation System using TruSeq PE Cluster Kit v4-cBot-HS (Illumia) according to the manufacturer’s instructions. After cluster generation, the library preparations were sequenced on an Illumina platform and paired-end reads were generated. KOBAS software was used to test the statistical enrichment of differential expression genes in KEGG pathways (Mao et al., 2005).

### Description of *S. aeria*

*S. aeria* were obtained from the feces of a patient under HTHH conditions. The results of 16S rDNA identification are shown in the **Supplementary Materials**.

### *S. aeria* Preparation

*S. aeria* were cultured in Yeast Mold Broth (YM broth) at 37°C for 18 hours, then fungal cells were concentrated *via* centrifugation at 14,000 rpm for 15 min and washed twice with sterilized phosphate-buffered saline (Ph 7.2). OD values were measured in triplicate at 600 nm, and the average value was used. The fungal cells were diluted to a final dose of 7.0 × 10^8^ CFU/mL (OD 600 = 1 was approximately equal to a bacterial concentration of 1.0 × 10^8^ CFU/mL).

### Statistical Analysis

Data are described as means ± the standard deviation of at least three independent experiments. Datasets that involved more than two groups were analyzed *via* one-way analysis of variance using Statistical Package for the Social Sciences software (SPSS version 17.0, Abacus Concepts, Berkeley, CA, USA) or Prism8 software (GraphPad, San Diego, CA, USA, **Table 5**).

**Table 5:** Software and Algorithms.

Reagent or Resource	Source	Identifier
GraphPad Prism 8.0.1	GraphPad Software	https://www.graphpad.com/
R version 4.0.1	Bell Laboratories (formerly AT&T, now Lucent Technologies)	https://www.r-project.org/

## Results

### Colonic Mycobiota Dysbiosis Under HTHH Conditions

In alpha diversity analysis of colonic mycobiota the indexes of Shannon differed significantly (**Figure 1B**, **Table 6**), and in principal component analysis there were differences between the control group and the experimental group (**Figure 1C**). Colonic mycobiota changes were assessed *via* the ITS1 technique. A histogram of species distribution at the levels of order, family, genus, and species are shown in **Figure 1D**. The relative abundances of *Zygosaccharomyces*, *Wickerhamomyces*, *Starmerella*, *Stagonospora*, *Pichia*, *Sporobolomyces*, *Rhodotorula*, *Rhizophagus*, *Purpureocillium*, *Plectosphaerella*, *Penicillium*, *Mortierella*, *Kodamaea*, *Kazachstania*, *Hanseniaspora*, *Fusarium*, *Filobasidium*, *Candida*, and *Botryotrichum* were increased (**Figure 1D**, **Table 7**, and **Figure S1**). The species that changed most significantly are shown in **Figure 1E**. Histograms of species distribution indicate that HTHH conditions altered the colonic mycobiota extensively.

**Table 6:** Microbial diversity index $\left(\bar{x}\;\pm \;SD\right)$.

Group	ACE	Chao1	Simpson	Shannon
Control	475.2 ± 26.3	477.2 ± 26.5	0.8765 ± 0.0055	4.3248 ± 0.097
Model	468.1 ± 50.2	474.5 ± 50.0	0.9738 ± 0.0023	6.7435 ± 0.0575

**Table 7:** 71 fungus at genus level in normal group were changed by the condition of the high temperature and high humidity (*p* value <0.01).

Genus	Changes in abundance	Modify	Genus	Changes in abundance	Modify
*Penicillium*	7.8869%	up	*Pseudogymnoascus*	0.0958%	up
*Fusarium*	6.3765%	up	*Paraglomus*	0.0804%	up
*Mortierella*	4.1440%	up	*Hannaella*	0.0763%	up
*Plectosphaerella*	1.7074%	up	*Malassezia*	0.0751%	up
*Tausonia*	1.6460%	up	*Microdochium*	0.0738%	up
*Humicola*	1.4352%	up	*Exophiala*	0.0735%	up
*Rhizophagus*	1.0118%	up	*Mycosphaerella*	0.0722%	up
*Solicoccozyma*	0.9925%	up	*Stropharia*	0.0720%	up
*Trichoderma*	0.9365%	up	*Septoria*	0.0691%	up
*Lecanicillium*	0.8303%	up	*Botrytis*	0.0687%	up
*Alternaria*	0.8222%	up	*Nigrospora*	0.0651%	up
*Fusicolla*	0.7223%	up	*Phialophora*	0.0620%	up
*Acrocalymma*	0.7166%	up	*Conlarium*	0.0563%	up
*Purpureocillium*	0.6670%	up	*Leptosphaeria*	0.0527%	up
*Botryotrichum*	0.6629%	up	*Thelonectria*	0.0380%	up
*Cladosporium*	0.5244%	up	*Conocybe*	0.0373%	up
*Clonostachys*	0.4180%	up	*Knufia*	0.0317%	up
*Leucoagaricus*	0.4045%	up	*Achroiostachys*	0.0296%	up
*Coniochaeta*	0.3746%	up	*Paraphoma*	0.0293%	up
*Thielavia*	0.3625%	up	*Clitopilus*	0.0288%	up
*Trichosporon*	0.2555%	up	*Cyphellophora*	0.0269%	up
*Chaetomium*	0.2544%	up	*Didymella*	0.0207%	up
*Staphylotrichum*	0.2478%	up	*Peziza*	0.0148%	up
*Corynascella*	0.2403%	up	*Saccharomyces*	0.0107%	up
*Microascus*	0.2362%	up	*Sterigmatomyces*	-0.0113%	down
*Marasmius*	0.1931%	up	*Stagonospora*	-0.0130%	down
*Codinaea*	0.1916%	up	*Kodamaea*	-0.0145%	down
*Filobasidium*	0.1845%	up	*Hyphopichia*	-0.0165%	down
*Panaeolus*	0.1692%	up	*Sporobolomyces*	-0.0309%	down
*Stachybotrys*	0.1491%	up	*Rhodotorula*	-0.0368%	down
*Wallemia*	0.1239%	up	*Hanseniaspora*	-0.2018%	down
*Papiliotrema*	0.1174%	up	*Kazachstania*	-0.5368%	down
*Polyschema*	0.1145%	up	*Starmerella*	-2.8127%	down
*Tetracladium*	0.1102%	up	*Wickerhamomyces*	-4.2380%	down
*Pichia*	-20.1428%	down	*Zygosaccharomyces*	-33.6231%	down
*Candida*	-2.5646%	down			

### HTHH Conditions and Appetite Regulation *via* the Gut-Brain Axis

**Figure 2:**
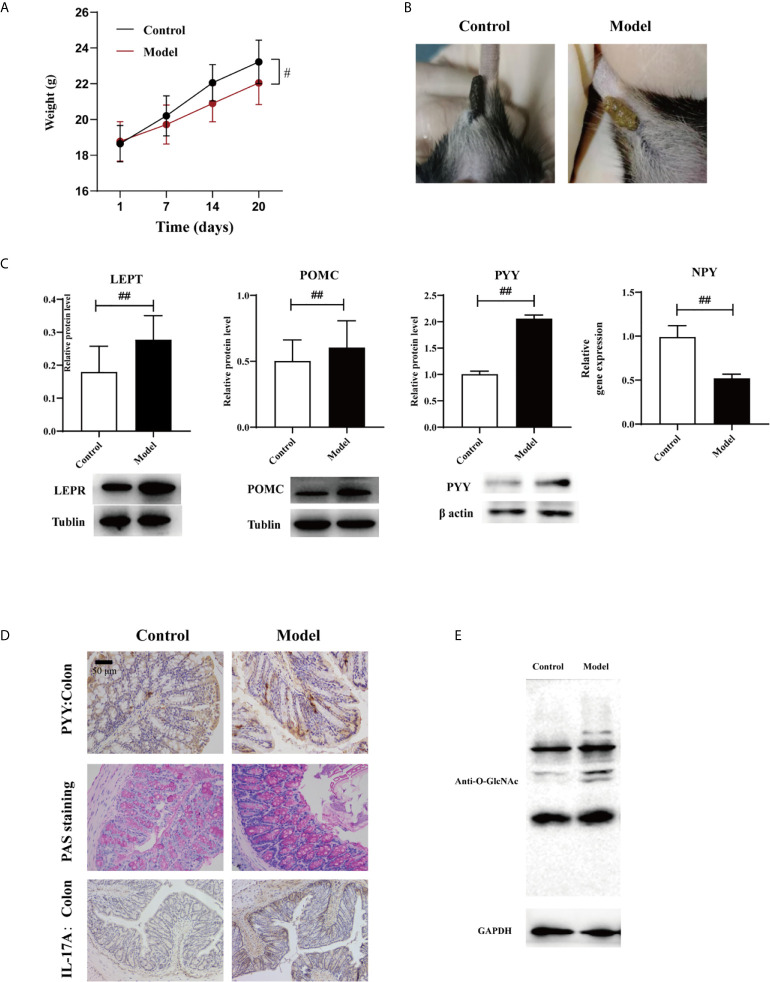
Effects of HTHH conditions on appetite in mice exerted *via* the gut-brain axis **(A)** Body weight in the experimental group was significantly lower than that in the control group. **(B)** The appearance and morphology of feces differed in the control group and the experimental group. **(C)** Western blotting results of LEPT and POMC expression in the hypothalamus, and PYY expression in the colon. NPY expression in the hypothalamus was detected *via* qPCR (*n* = 3, two-tailed Student’s *t*-test. ^#^p < 0.05, ^##^p < 0.01. **(D)** PYY and IL-17A expression detected *via* immunohistochemistry. Periodic acid–Schiff staining was used to detect glycogen and mucin in colon tissue. **(E)** GlcNAcylation in the colon was detected using a pan anti-O-GlcNAc monoclonal antibody (*n* = 3).

Body weight was lost and fecal traits were altered in the experimental group (**Figures 2A**, **B**). Expression levels of LEPT and POMC proteins in the hypothalamus determined *via* western blotting were altered in the experimental group, as was the expression of NPY determined *via* qPCR (**Table 8**), and the levels of all three were associated with leptin (**Figure 2C**, *p* < 0.01). This suggests that HTHH conditions may influence the central nervous system and negative feedback regulation of appetite and metabolism, and immunohistochemistry results exhibited the same trends (**Figure 2D**). PYY was activated in the colon (**Figures 2C, D**). GlcNAcylation was increased in the colon in the experimental group (**Figure 2E**, *p* < 0.05). Periodic acid–Schiff staining showed that glycogen and mucin were increased in colon tissues in the experimental group (**Figure 2D**).

**Table 8:** Sequence-Based Reagents.

Gene	Primer	Sequence (5’→3’)	Amplicon Size
NPY	Mus-NPY-F161	5’-ATGCTAGGTAACAAGCGAATGG-3’	161
	Mus-NPY-R161	5’-TGTCGCAGAGCGGAGTAGTAT-3’	
GAPDH	Mus-GAPDH-F117	5’-TCTCCTGCGACTTCAACA-3’	117
	Mus-GAPDH-R117	5’-TGTAGCCGTATTCATTGTCA-3’	

### Fecal Content Metabolomics and HTHH Conditions

In metabolic analysis of fecal contents in the experimental group 17 different metabolites were detected *via* GC/MS. Of these 17, D-glucose and maltose were downregulated whereas the other 15 were upregulated (**Figure S2A**, **Table 9**), including oleanitrile, stearic acid, octadecadienoic acid, aspartic acid, tromethamine, norleucine, alanine, glycine, cadaverine, oxoproline, hydroxy-L-norleucine, phosphonic acid, cholesterol, and fucose (**Figure S2A**, **Table 9**).

**Table 9:** 17 fecal metabolites which in normal group changed by the condition of the high temperature and high humidity (*p* value < 0.01).

Metabolite (M *vs* N)	Modify	Metabolite (M *vs* N)	Modify
Phosphonic acid	up	Cholesterol	up
Stearic acid	up	L-Aspartic acid	up
L-5-Oxoproline	up	Glycine	up
9,12-Octadecadienoic acid (Z,Z)-	up	Tromethamine	up
Oleanitrile	up	(-)-Fucose	up
L-Alanine	up	Cadaverine	up
2,6-Bis (tert-butyl)phenol	up	Maltose	down
6-Hydroxy-L-norleucine	up	D-glucose	down
L-Norleucine	up		

### Conjoint Correlational Analysis of Mycobiota Genus-Fecal Metabolites

Based on Spearman’s correlational analysis, correlation networks were generated using a probability threshold of *p* < 0.01 (the top 20 of changes in abundance) and a correlation coefficient threshold of > 0.7 (**Figures S2B, C**). The mycobiota genera *Candida*, *Starmerella*, *Wickerhamomyces*, *Pichia*, and *Zygosaccharomyces* were significantly associated with the metabolites phosphonic acid, D-glucose, stearic acid, L-5-oxoproline, 9,12-(Z, Z)-octadecadienoic acid, oleanitrile, L-alanine, 2,6-bis(tert-butyl)phenol, 6-hydroxy-L-norleucine, L-norleucine, cholesterol, L-aspartic acid, glycine, tromethamine, (-)-fucose, cadaverine, and maltose.

Some harmful metabolites were positively correlated with the abundance of fungus. Metabolites of cholesterol were positively correlated with *Penicillium*, *Fusarium*, *Mortierella*, *Plectosphaerell*a, *Purpureocillium*, *Botryotrichum*, and *Candida* (**Figures S2B, C**). Metabolites of stearic acid were positively correlated with *Penicillium*, *Fusarium*, *Mortierella*, *Plectosphaerella*, *Tausonia*, *Trichoderma*, *Solicoccozyma*, *Rhizophagus*, *Lecanicillium*, *Purpureocillium*, *Botryotrichum*, and *Candida* (**Figures S2B, C**). Metabolites of tromethamine were positively correlated with *Penicillium*, *Fusarium*, *Mortierella*, *Humicola*, *Plectosphaerella*, *Tausonia*, *Trichoderma*, *Solicoccozyma*, *Rhizophagus*, *Lecanicillium*, *Pichia*, *Candida*, *Acrocalymma*, *Wickerhamomyces*, *Starmerella*, *Zygosaccharomyces*, *Botryotrichum*, and *Purpureocillium* (**Figures S2B, C**). Metabolites of D-glucose were negatively correlated with *Penicillium*, *Fusarium*, *Mortierella*, *Plectosphaerella*, *Tausonia*, *Humicola*, *Rhizophagus*, *Solicoccozyma*, *Trichoderma*, *Lecanicillium*, *Alternaria*, *Fusicolla*, *Acrocalymma*, *Purpureocillium*, and *Botryotrichum* (**Figures S2B, C**). Metabolites of maltose were negatively correlated with *Penicillium*, *Fusarium*, *Mortierella*, *Plectosphaerella*, *Rhizophagus*, *Purpureocillium*, and *Botryotrichum* (**Figures S2B, C**).

### *S. aeria* Was Higher in the Saliva of Humans Under HTHH Conditions

**Figure 3:**
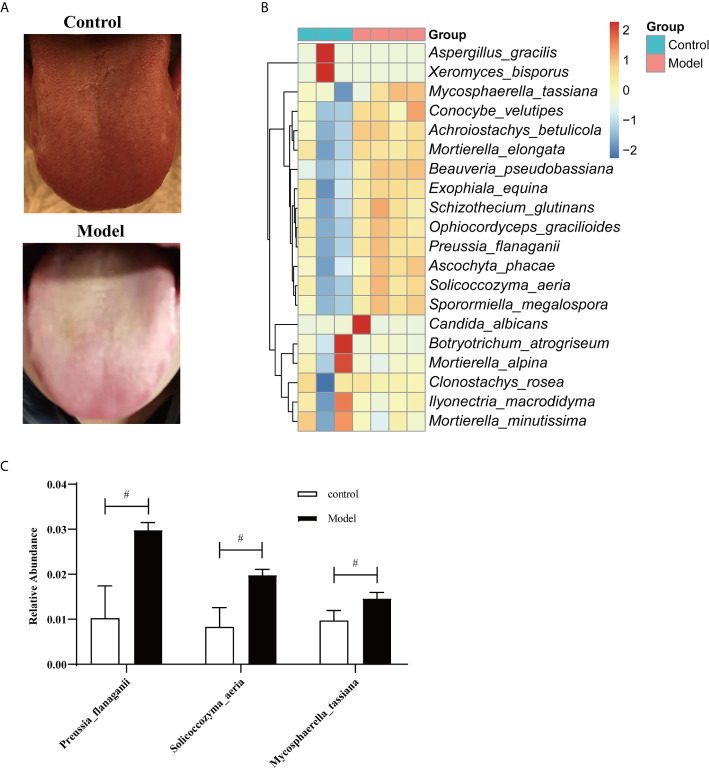
Fungal microbiome in saliva from humans during HTHH conditions **(A)** The appearance and morphology of the tongue coatings are different. **(B)** Heatmap of the fungal microbiome at the species level. The x-coordinate represents species, and the 20 species with the lowest *p* values are shown. The ordinate represents the relative abundance of species. Columns of different colors represent each sample. **(C)** The three with the greatest changes in abundance at the species level (*t*-test). ^#^p < 0.05.

Tongue coatings were thick and greasy during HTHH conditions (**Figure 3A**). A species-level heatmap is shown in **Figure 3B**. *Mycosphaerella tassiana*, *Conocybe velutipes*, *Achroiostachys betulicola*, *Morietella elongate*, *Beauveria pseudobassiana*, *Exophiala equina*, *Schizothecium glutinans*, *Ophiocordyceps gracilioides*, *Preussia flanaganii*, *Ascochyta phacae*, *Solicoccozyma aeria*, and *Sporormiella megalospora* were increased, indicating that the saliva mycobiota was significantly altered. The three most significantly affected species are shown in **Figure 3C**. *S. aeria* was significantly altered in humans and mice.

### *S. aeria*-Associated Appetite Suppression and IL-17R Pathway Activation in Mice

**Figure 4:**
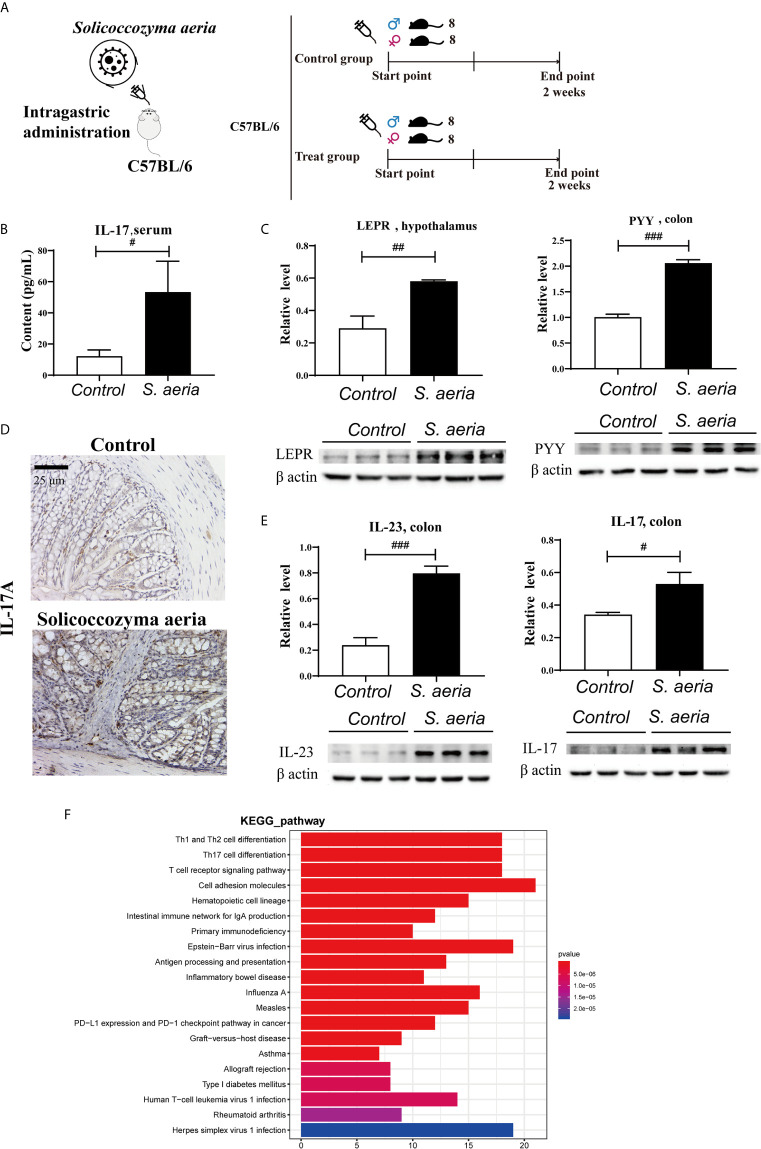
Appetite suppression and IL17RA pathway activation by *S. aeria* gavage **(A)** Schematic diagram of the *S. aeria* gavage experiment. **(B)** Changes in serum IL-17 determined using an enzyme-linked immunosorbent assay kit (*n* = 6, two-tailed Student’s *t*-test). **(C)** PYY expression levels in colon tissues, and LEPT expression levels in the hypothalamus determined *via* western blotting (*n* = 3, two-tailed Student’s *t*-test). **(D)** IL-17A expression levels in colon tissues determined *via* immunohistochemistry. **(E)** IL-23 and IL-17A expression levels in colon tissues determined *via* western blotting (*n* = 3, two-tailed Student’s *t*-test). ^#^ *p* < 0.05, ^##^ *p* < 0.01, ^###^ *p* < 0.001 **(F)** Enrichment analysis of colonic differentially expressed genes in KEGG pathway Note: The abscisate is the number of genes of interest annotated in this entry, and the ordinate is each pathway entry. The color of the column represents the P value of the hypergeometric test.

In western blotting analyses LEPT and PYY protein levels were upregulated in the treatment group (**Figure 4C**, *p* < 0.05). This suggests that *S. aeria* may influence the central nervous system, and negative feedback regulation of appetite. Serum levels of IL-17 (**Figure 4B**, *p* < 0.05), and colon tissue expression levels of IL-23 and IL-17 were significantly upregulated (**Figures 4D, E**, *p* < 0.05). The IL-17RA pathway may be activated by gut microbiota (**Figure 4F**), and is related to most autoimmune diseases, such as rheumatoid arthritis, systemic lupus erythematosus, ankylosing spondylitis (AS), and inflammatory bowel disease (Atarashi et al., 2015; Kim et al., 2016). IL-23 may induce pathogenic Th17 cells and promote inflammatory disease (Lee et al., 2020).

### *S. aeria* and Gut Microbiota in C57BL/6 Mice

**Figure 5:**
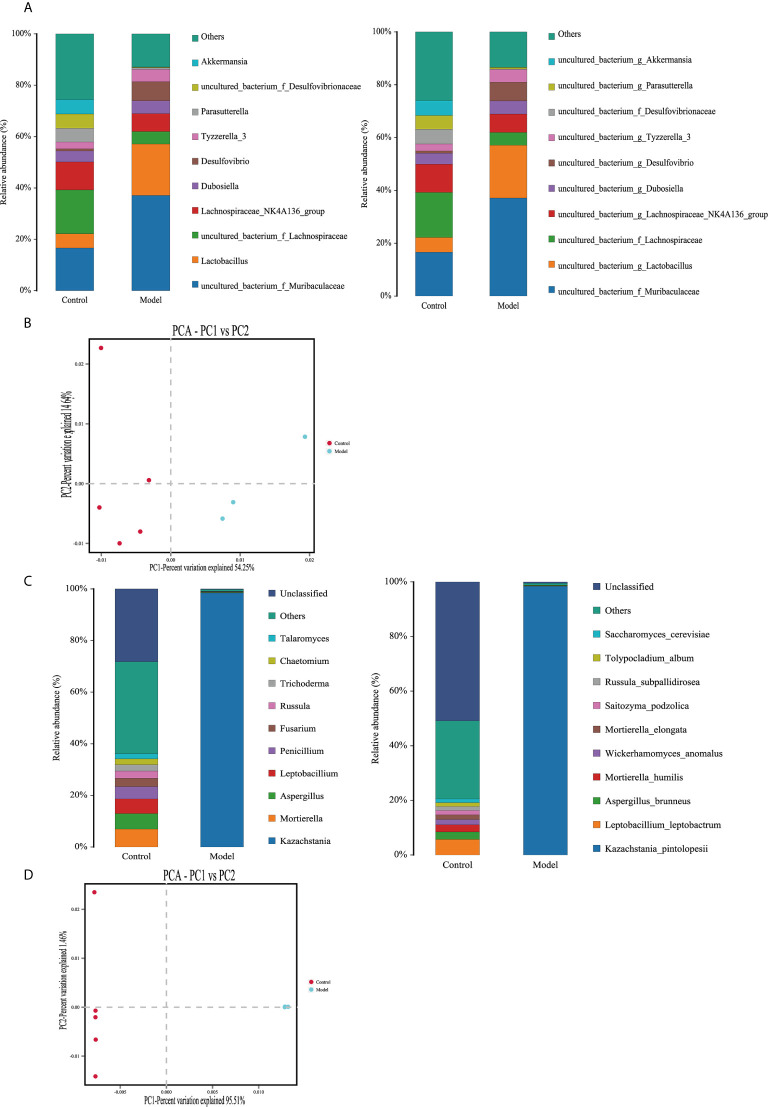
Changes in the bacterial microbiomes and fungal microbiomes in the treatment group and the control group **(A)** Histogram of species distribution in the bacterial microbiome (left, genus level; right, species level). **(B)** Principal component analysis. **(C)** Histogram of species distribution in the fungal microbiome (left, genus level; right, species level). **(D)** Left, principal component analysis.

In 16S rDNA sequencing analyses at the genus level the relative abundances of *Bacteroidales*, *Lactobacillales*, *Desulfovibrionales*, *Saccharimonadales*, and *Erysipelotrichales* were increased in the *S. aeria*-treated mice, whereas those of *Clostridiales*, *Verrucomicrobiales*, *Betaproteobacteriales*, *Coriobacteriales*, and *Campylobacterales* were reduced (**Figure 5A**, left). At the species level the relative abundances of *Muribaculaceae*, *Lactobacillus*, *Desulfovibrio*, *Tyzzerella 3*, and *Dubosiella* were increased, whereas those of *Lachnospiraceae*, *Akkermansia*, *Desulfovibrionaceae*, *Parasutterella*, and *Lachnospiraceae NK4A136* group were reduced (**Figure 5A**, right). In principal component analysis there were differences between the control group and the treated group (**Figure 5B**).

In ITS sequencing analyses, at the genus level in the *S. aeria*-treated group the relative abundance of *Kazachstania* was increased, whereas those of *Talaromyces*, *Chaetomium*, *Trichoderma*, *Russula*, *Fusarium*, *Penicillium*, *Leptobacillium*, *Aspergillus*, and *Mortierella* were reduced (**Figure 5C**, left). At the species level *Kazachstania pintolopesii* was significantly increased, whereas *Leptobacillium leptobactrum*, *Mortierella humilis*, *Aspergillus brunneus*, *Wickerhamomyces anomalus*, *Mortierella elongata*, *Saitozyma podzolica*, *Russula subpallidirosea*, *Tolypocladium album*, and *Saccharomyces cerevisiae* were reduced (**Figure 5C**, right). In principal component analysis there were differences between the control group and the treated group (**Figure 5D**).

In previous studies enrichment of *Escherichia-Shigella* (Proteobacteria), *Veillonella* (Firmicutes), *Faecalibacterium* (Firmicutes), *Eubacterium rectale* group (Firmicutes), *Streptococcus* (Firmicutes), *Lachnospiraceae NK4A136* group (Firmicutes), and reduced *Prevotella strain 9* (Bacteroidetes), *Megamonas* (Firmicutes), and *Fusobacterium* (Fusobacteria) were detected in AS patients (Li et al., 2019). AS-enriched species including *Bacteroides coprophilus*, *Parabacteroides distasonis*, *Eubacterium siraeum*, *Acidaminococcus fermentans* and *Prevotella copri* were identified in AS patients *via* metagenomic analyses (Zhou et al., 2020). *Desulfovibrionales* is the main producer of hydrogen sulfide in the intestinal tract. High concentrations of hydrogen sulfide are associated with intestinal inflammation, and *Desulfovibrionales* plays an important role in the occurrence and development of inflammatory bowel disease (Dordević et al., 2021; Kushkevych et al., 2021).

*Lachnospiraceae NK4A136* group, *Desulfovibrionales*, and *Bacteroidales* were increased in treatment group. *Desulfovibrionales* could produce toxic H_2_S and have a strong association with IBD (Inness et al., 2007), and that *S. aeria* is a powerful trigger in some susceptible hosts. *S. aeria* significantly altered the abundance of *K. pintolopesii*. It’s reported that *C.albians* is not a natural colonizer of mucosal surfaces in these-the rodent equivalent of normal flora yeast is *K.pintolopesii* (Naglik et al., 2008), thus we speculate that *K. pintolopesii* may be a conditional pathogen which activate the IL17A pathway.

## Discussion

Illness-associated behaviors such as loss of appetite, fatigue, low-grade fever, drowsiness, and/or chills usually indicate pathogenic infection and/or low immunity. In the current study food intake and body weight were reduced, and in the hypothalamus the appetite-related proteins LEPT and POMC were upregulated (*p* < 0.05) and *NPY* gene expression was downregulated (**Figure 2**, *p* < 0.05). In colonic tissues the expression levels of PYY and O-GlaNAc were altered, as was periodic acid–Schiff staining (**Figure 2**, *p* < 0.05). IL-17 expression was upregulated in the colon, indicating that in mice appetite and immunity were altered under HTHH conditions. Other colonic mycobiota and fecal metabolome parameters were also significantly altered under HTHH conditions (**Figures 1** and **2**), indicating that HTHH can change the mycobiota and metabolome, then influence host behaviors.

Little attention has been paid to autoimmune diseases triggered by weather or high humidity. Few studies have investigated relationships between humidity-induced microbial alterations and autoimmune diseases, and no causative characteristic microbe has been reported. In the present study environmental temperature and humidity could trigger homeostasis changes in human microbes, especially fungi, activate IL17A pathway and elevate the level of IL17A in serum. There was also significant elevation of the characteristic fungi *S. aeria*, and that species was present at high relative abundance in the HTHH mice and humans. Appetite suppression and IL-17R signaling activation were detected in mice, as were disturbances of the gut microbiota and mycobiota. *S. aeria* may trigger *K. pintolopesii* proliferation in some susceptible hosts, but this requires extensive further investigation.

In previous studies germ-free conditions or limited microbiota content attenuated arthritis severity in the ZAP70^W163C^ BALB/c (SKG) mouse model (Rehaume et al., 2014), and spondylitis and colitis disappear when breeding in the GF state in the HLA-B27 transgenic mouse model (Taurog et al., 1994). These observations indicate that microbes play an important role in autoimmune diseases. Th17 cells and IL-17 are involved in host defenses by way of immunothrombosis, and promote the removal of pathogenic microorganisms (Amezcua Vesely et al., 2019; Kitamoto et al., 2020), but too much IL-17A is fatal for the host, and can lead to a variety of inflammatory or autoimmune diseases (Gravallese and Schett, 2018; Jo et al., 2018).

It is not clear what substances cause the activation of autoimmune disease and activate the IL-17RA pathway, or whether *S. aeria* is significantly increased during the onset of inflammatory bowel disease, rheumatoid arthritis, AS and other autoimmune diseases. HTHH conditions can lead to imbalanced gut and oral microbiomes, particularly significant changes in fungi, which provides a new perspective with respect to the study of microbe-related diseases induced by climate changes. The microbial diversity hosted by specific pathogen-free mice is relatively simple, and the microbial diversity hosted by wild mice is reportedly completely different, which imposes substantial limitations on the study of interactions and functions of the relevant microbes in the natural environment (Rosshart et al., 2019). In the future, germ-free and ZAP70^W163C^ BALB/c (SKG) mice will be used to test the effects of *S. aeria* and *K. pintolopesii*.

## Data Availability Statement

The datasets presented in this study can be found in online repositories. The names of the repository/repositories and accession number(s) can be found in the article/**Supplementary Material**.

## Ethics Statement

The studies involving human participants were reviewed and approved by Guangdong Second Provincial General Hospital, Guangzhou 510000, Guangdong, China. Written informed consent to participate in this study was provided by the participants’ legal guardian/next of kin. The animal protocols used in this study were approved by the Institutional Animal Care and Use committee of the Center of Laboratory Animals of the Guangdong Institute of Microbiology.

## Author Contributions

DC and JW designed this study. YG wrote the manuscript. YG and HG collected animal physiological data and fecal samples, and did the physiological and biochemical indexes measurement. YG and XZ did the western blotting analysis. YG did the metabolomic analysis. YG collected data regarding the microbial metabolic networks analysis. DC and JW helped to design the study and to develop the multi-omics analysis methods, reviewed this manuscript and offer all the necessary research start-up fund, experimental platform. All authors contributed to the article and approved the submitted version.

## Funding

The present work was supported by the financial support from the National Natural Science Foundation of China (81941010 and 8187151662), Guangzhou Science and Technology Plan Projects (2017A070701003, 2017B020231001, and 2019A030317009), GDAS’ Project of Science and Technology Development (2019GDASYL-0104007), and Project of Science and Technology Development of Guangdong Second Provincial General Hospital (YY2019-006).

## Conflict of Interest

The authors declare that the research was conducted in the absence of any commercial or financial relationships that could be construed as a potential conflict of interest.

## Supplementary Material

Supplementary Figure 1 Species-level heatmap Species-level heatmap generated using sample abundance similarity for clustering. The difference between two samples is represented visually by the change in color gradient.

Supplementary Figure 2 **(A)** Heatmap of significant metabolites depicting differences between the control group and the experimental group. **(B)** Conjoint analysis of 71 significantly different fungal genera and 17 significantly different fecal metabolites (+*p* < 0.1, ++*p* < 0.05, +++ *p* < 0.01). **(C)** Spearman’s correlational coefficients for different fungal genera (top 20 with respect to changes in abundance) and 17 significantly different fecal metabolites. In the correlational analysis the probability threshold used to construct correlation networks was *p* < 0.01, and the correlation coefficient threshold used was > 0.7. Blue nodes represent metabolites, and yellow nodes represent fungal genera. The red line indicates a positive correlation and the black line indicates a negative correlation. More nodal connections indicates more associations with others, and the sizes of nodes correspond with the change in abundance.

Supplementary Figure 3 Fecal content metabolomics were altered under HTHH conditions **(A)** Principal component analysis, PLS-DA analysis, and OPLS-DA analysis of fecal metabolites. **(B)** Pathway analysis of fecal metabolites in the control group and the experimental group.
