# Supplementary figures and images for: Appetite Suppression and Interleukin 17 Receptor Signaling Activation of Colonic Mycobiota Dysbiosis Induced by High Temperature and High Humidity Conditions
Source: Front Cell Infect Microbiol. 2021 Sep 10;11:657807. doi: 10.3389/fcimb.2021.657807 (PMC8462304; doi:10.3389/fcimb.2021.657807)

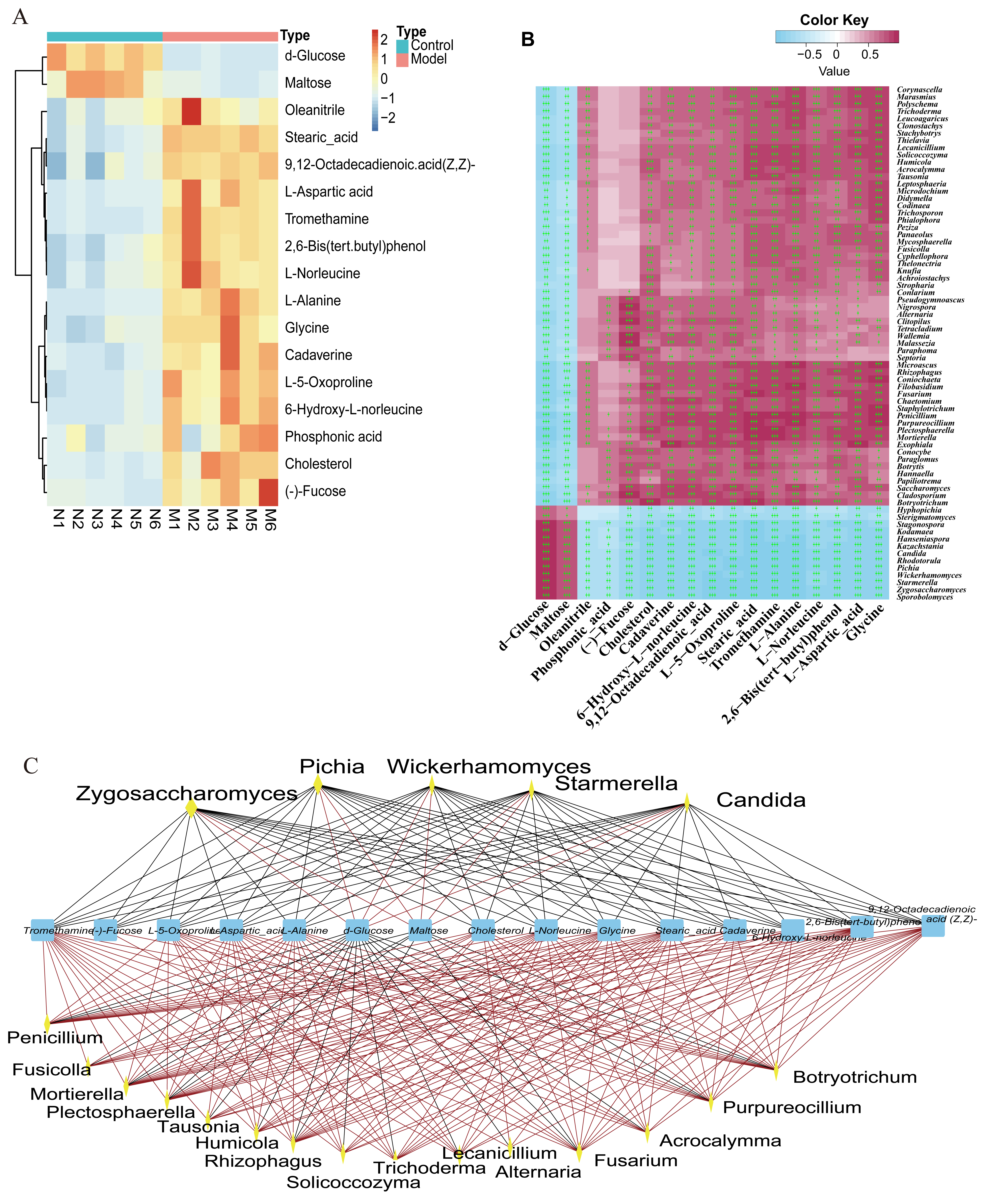

Supplement: Supplementary Figure 2 — (A) Heatmap of significant metabolites depicting differences between the control group and the experimental group. (B) Conjoint analysis of 71 significantly different fungal genera and 17 significantly different fecal metabolites (+p < 0.1, ++p < 0.05, +++ p < 0.01). (C) Spearman’s correlational coefficients for different fungal genera (top 20 with respect to changes in abundance) and 17 significantly different fecal metabolites. In the correlational analysis the probability threshold used to construct correlation networks was p < 0.01, and the correlation coefficient threshold used was > 0.7. Blue nodes represent metabolites, and yellow nodes represent fungal genera. The red line indicates a positive correlation and the black line indicates a negative correlation. More nodal connections indicates more associations with others, and the sizes of nodes correspond with the change in abundance. [file Image_2.tif]
